# Serial crystallography with multi-stage merging of thousands of images

**DOI:** 10.1107/S2053230X22006422

**Published:** 2022-07-04

**Authors:** Alexei S. Soares, Yusuke Yamada, Jean Jakoncic, Sean McSweeney, Robert M. Sweet, John Skinner, James Foadi, Martin R. Fuchs, Dieter K. Schneider, Wuxian Shi, Babak Andi, Lawrence C. Andrews, Herbert J. Bernstein

**Affiliations:** a Center for BioMolecular Structure, Brookhaven National Laboratory, Upton, New York, USA; b High Energy Accelerator Research Organization (KEK), Tsukuba, Ibaraki, Japan; c University of Bath, Bath, United Kingdom; d Ronin Institute for Independent Scholarship, Kirkland, Washington, USA; e Ronin Institute for Independent Scholarship, c/o National Synchrotron Light Source II, Building 745, Brookhaven National Laboratory, Upton, New York, USA

**Keywords:** clustering, serial crystallography, *KAMO*, *BLEND*

## Abstract

*KAMO* and *BLEND* provide particularly effective tools to automatically manage the merging of large numbers of data sets from serial crystallography. The requirement for manual intervention in the process can be reduced by extending *BLEND* to support additional clustering options such as the use of more accurate cell distance metrics and the use of reflection-intensity correlation coefficients to infer ‘distances’ among sets of reflections. This increases the sensitivity to differences in unit-cell parameters and allows clustering to assemble nearly complete data sets on the basis of intensity or amplitude differences. If the data sets are already sufficiently complete to permit it, one applies *KAMO* once and clusters the data using intensities only. When starting from incomplete data sets, one applies *KAMO* twice, first using unit-cell parameters. In this step, either the simple cell vector distance of the original *BLEND* or the more sensitive NCDist is used. This step tends to find clusters of sufficient size such that, when merged, each cluster is sufficiently complete to allow reflection intensities or amplitudes to be compared. One then uses *KAMO* again using the correlation between reflections with a common *hkl* to merge clusters in a way that is sensitive to structural differences that may not have perturbed the unit-cell parameters sufficiently to make meaningful clusters. Many groups have developed effective clustering algorithms that use a measurable physical parameter from each diffraction still or wedge to cluster the data into categories which then can be merged, one hopes, to yield the electron density from a single protein form. Since these physical parameters are often largely independent of one another, it should be possible to greatly improve the efficacy of data-clustering software by using a multi-stage partitioning strategy. Here, one possible approach to multi-stage data clustering is demonstrated. The strategy is to use unit-cell clustering until the merged data are sufficiently complete and then to use intensity-based clustering. Using this strategy, it is demonstrated that it is possible to accurately cluster data sets from crystals that have subtle differences.

## Abstract 2

The effectiveness of clustering in merging data sets from large numbers of crystals in serial crystallography can be improved by combining multiple clustering techniques using unit-cell parameter-based clustering for very incomplete sets and switching to reflection-based clustering once the preliminary merging has increased the completeness.

## 1. Introduction

**Figure 1:**
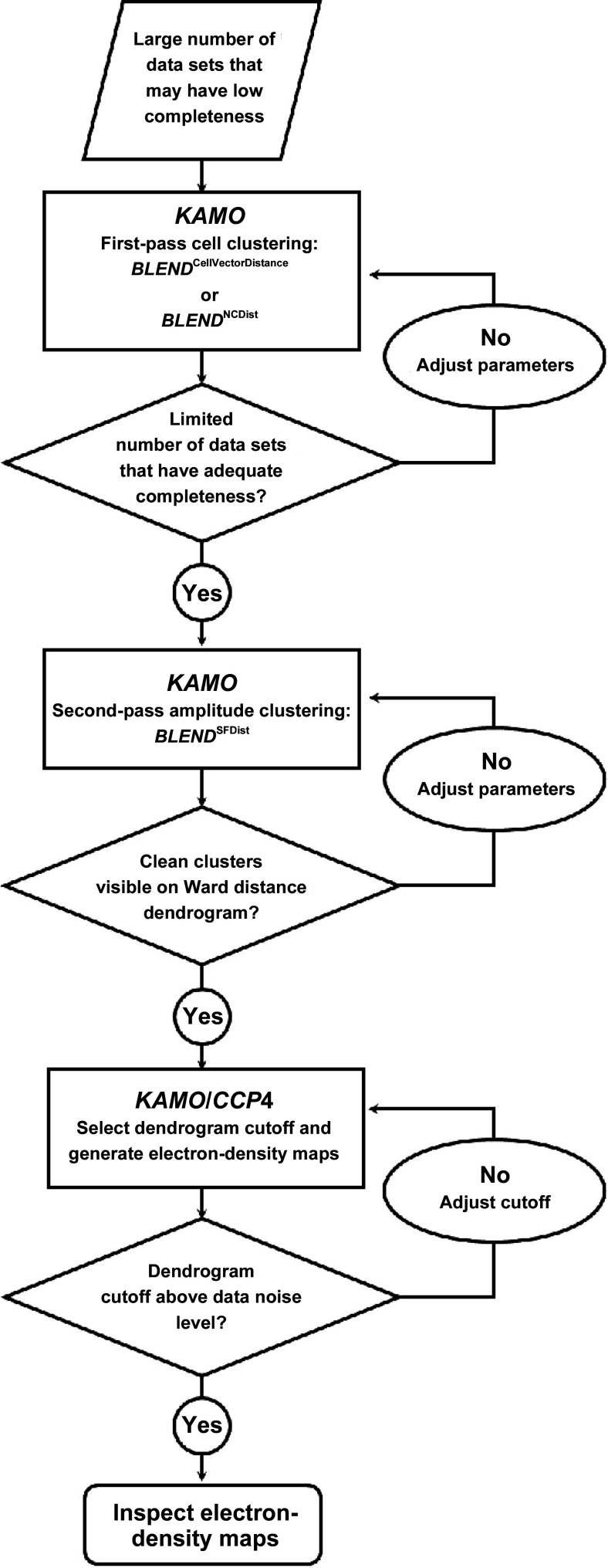
Process flow in the use of *KAMO* and *BLEND*. In the case of the four-way clustering discussed in Sections 3 and 4, a total of 896 data sets were input to the first-stage NCDist clustering engine and a total of 107 data sets were input to the second-stage SFDist clustering engine (first and second rectangles).

*KAMO* (Yamashita, 2017 ▸; Yamashita *et al.*, 2017 ▸, 2018 ▸; Hasegawa *et al.*, 2017 ▸) and *BLEND* (Foadi *et al.*, 2013 ▸) provide particularly effective tools to automatically manage the merging of large numbers of data sets from serial crystallo­graphy. The requirement for manual intervention in the process can be reduced by extending *BLEND* to support additional clustering options, thereby increasing the sensitivity to differences in unit-cell parameters and allowing clustering to assemble nearly complete data sets on the basis of intensity or amplitude differences. *KAMO* provides the necessary process-flow management infrastructure. This process flow is shown in Fig. 1 ▸. If the data sets are already sufficiently complete to permit it, one applies *KAMO* once and clusters the data using intensities only. When starting from incomplete data sets, one applies *KAMO* twice, first using unit-cell parameters. In this step, either the simple cell vector distance of the original *BLEND* or the more sensitive NCDist is used to find clusters to merge to achieve sufficient completeness to allow intensities or amplitudes to be compared. One then uses *KAMO* again using the correlation between the reflections with common *hkl* indices (Assmann *et al.*, 2016 ▸) to merge clusters in a way that is sensitive to structural differences that may not have perturbed the unit-cell parameters sufficiently to make meaningful clusters.

X-ray free-electron lasers (XFELs) have pioneered effective crystallographic data collection from large numbers of crystals (Colella & Luccio, 1984 ▸; Neutze *et al.*, 2000 ▸). Serial crystallography, an essential technique at XFEL light sources, has become an important technique at synchrotrons (Giordano *et al.*, 2012 ▸; Liu *et al.*, 2013 ▸; Rossmann, 2014 ▸; Standfuss & Spence, 2017 ▸), especially at newer high-intensity synchrotron beamlines (Pearson & Mehrabi, 2020 ▸). The data may be organized either as XFEL-like still images or as thousands of wedges of data produced from very large numbers of crystals. The stills and wedges then need to be carefully organized into reasonably homogeneous clusters of data that can be merged for processing. This will become one of the common tools to assemble complete data from many partial wedges in molecular replacement, SAD and ligand studies and to sort classes of crystals for studies of dynamics, binding, interactions *etc.* *KAMO* includes cluster analysis based both on unit-cell parameters and on reflection correlation coefficients. The clustering is based on Ward’s method, in which

Ward’s minimum variance criterion minimizes the total within-cluster variance. To implement this method, at each step find the pair of clusters that leads to minimum increase in total within-cluster variance after merging. This increase is a weighted squared distance between cluster centers.

(from https://en.wikipedia.org/wiki/Ward%27s_method).

In this paper, we discuss the issues involved in improving the sensitivity of both approaches to clustering, using as an example 999 5° wedges from lysozyme in four forms:

(i) NAG [native with *N*-acetylglucosamine (NAG) soaked in],
(ii) benzamidine (with benzamidine soaked in),
(iii) benzamidine plus NAG (native with both NAG and benzamidine soaked in) and
(iv) native (no ligands).

As we will see, although the unit-cell parameters are changed sufficiently to allow recognition of the NAG soak, it is difficult to filter the benzamidine soak simply on the basis of unit-cell parameter changes, suggesting the desirability of switching from cell-based clustering to intensity-based clustering as early in the process as possible. Hence, cell-based clustering is more universal, in the sense that we can successfully apply it very early in the structure-solution process, but it is less granular, in the sense that it cannot see as much detail as intensity-based clustering. Cell-based clusters are less able to discriminate between similar but non-identical forms.

Two-stage clustering can be regarded as a method to reduce the data multiplicity needed to achieve a desired level of data precision. Hence, it is a continuation of previous techniques (White *et al.*, 2012 ▸) that reduced the need for multiplicity compared with the Monte Carlo method of integration (Kirian *et al.*, 2010 ▸), which makes no assumptions regarding crystal-to-crystal scaling (and hence relies entirely on statistical averaging to achieve data precision from millions of observations).

An alternative to consider, rather than staging the cell-based clustering first and then applying intensity-based clustering, would be to combine the two approaches in a single stage of higher dimensionality. There are two problems that will have to be addressed in order to create such a single-stage combined algorithm.

Firstly, it is not possible to apply intensity-based clustering at all without first indexing all reflections in a way that provides a common unambiguous label for each reflection in each image to correctly identify corresponding reflections with the same index in each different image. One option to satisfy this requirement is to index all diffraction images in *P*1 or some other low symmetry common to all images. This may require more images and more reflections than are available and, worse, because symmetry is being ignored, may bring together for merging images describing very different molecular conformations.

Secondly, as the number of independent parameters increases, all clustering methods work increasingly poorly due to the ‘curse of dimensionality’ (Bellman, 1956 ▸). Combining the very well behaved low-dimensionality cell-based clustering with the high-dimensionality intensity-based clustering can obscure the results of cell-based clustering. In most cases, unless the information to be gained from cell-based clustering is available *a priori*, it is more useful to process the cell-based clustering information first and then to move on to intensity-based clustering in a second stage based on the cell-based results.

For cases in which the indexing is too ambiguous to reliably start the process, the approach used in *dials.cosym* should be considered (Gildea & Winter, 2018 ▸), which uses the averages of intensities within images and from multiple images simultaneously with spot positions for indexing, dealing with the issue of the curse of dimensionality by using the averaging to limit the increase in dimension as much as possible (Brehm & Diederichs, 2014 ▸; Diederichs, 2017 ▸). This is not the same as simultaneously performing full cell-based and full intensity-based clustering, which is not recommended.

## 2. Limits of conventional clustering

Since our goal was to expand the capabilities of existing clustering techniques, we began by applying a conventional clustering strategy to diffraction data from lysozyme microcrystals containing various combinations of known small-molecule binders. Microcrystals were preferred to avoid the conflation of structurally anisotropic data that has been demonstrated in larger crystals; see Thompson *et al.* (2018 ▸).

Lysozyme microcrystals suitable for acoustic harvesting (Soares *et al.*, 2011 ▸) were grown using batch crystallization by dissolving 120 mg ml^−1^ lysozyme in 0.2 *M* sodium acetate pH 4.6 (Hampton Research, catalog No. HR7-110) and combining it with an equal amount of precipitant (10% ethylene glycol + 12% sodium chloride) (Roessler *et al.*, 2016 ▸). The resulting slurry of 5–10 µm crystals was divided into four aliquots. Three of the four aliquots were then equilibrated overnight with an equal volume of 0.5 *M* solutions of benzamidine, NAG and benzamidine plus NAG. These two small molecules are known to bind tetragonal lysozyme crystals (Yin *et al.*, 2014 ▸). The fourth aliquot was diluted with an equal volume of mother liquor but contained no ligands.

The diffusion rate for benzamidine and NAG within lysozyme crystals is approximately 1 µm s^−1^ (Cole *et al.*, 2014 ▸). To prevent the cross-contamination of crystals with neighboring forms, crystals could not be mixed with different forms for more than 1 s before halting diffusion by plunge cryocooling in liquid nitrogen. To accomplish this, we deposited 5 µl of crystal slurry from each aliquot onto a separate agarose support (Cuttitta *et al.*, 2015 ▸). We used acoustic sound pulses to harvest 2.5 nl of crystal slurry from each of the four lysozyme aliquots and separately positioned them on a micro-mesh (MiTeGen, catalog No. M3-L18SP-10) so that none of the droplets was in contact with any other. Crystal-containing droplets were threaded through small apertures to prevent cross-contamination (Foley *et al.*, 2016 ▸). We then swept the non-crystal-containing side of the micro-mesh against a sponge moistened with cryoprotectant (mother liquor + 20% glycerol) and, in one smooth motion, immediately cryocooled the micro-mesh in liquid nitrogen. In addition to cryoprotection, this also mixed the crystals together into one contiguous field. The same procedure was repeated for a micro-mesh containing only two lysozyme forms: benzamidine plus NAG and native. Serial diffraction data were then obtained in 5° wedges from approximately 100 crystals on each micro-mesh.

**Figure 2:**
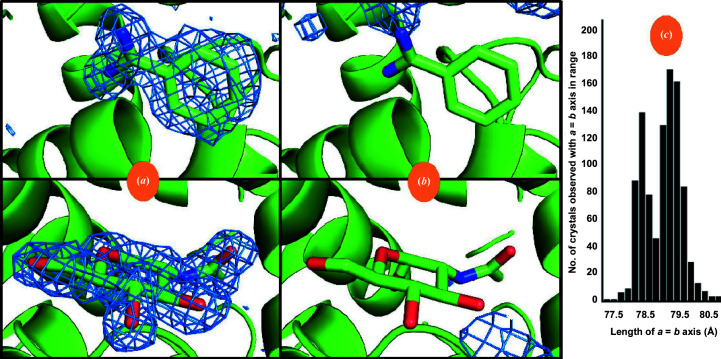
Electron-density maps calculated after two-way clustering of diffraction data obtained from micro-meshes that contained a mixture of doubly bound crystals (benzamidine plus NAG) (*a*) and native crystals (no ligands) (*b*). The omit difference maps are contoured at 1.5σ in the region expected to contain benzamidine (top) and NAG (bottom). The histogram cluster in (*c*) represents the unit-cell dimensions of the cluster of crystal data sets that yielded the omit difference map shown in (*a*). Similarly, the histogram cluster on the right in (*c*) represents the unit-cell dimensions of the cluster of crystal data shown in (*b*). Clearly the clustering algorithm was able to accurately partition the data for this simple two-way split. See Section S1.

The *KAMO* software package was then used in the default configuration (in which data are clustered based only on unit-cell similarity) to partition the diffraction data from micro-meshes containing the four lysozyme forms into four clusters and to partition the diffraction data from micro-meshes containing two lysozyme forms into two clusters. Each cluster of data was merged and then phased using the known structure of lysozyme. Subsequently, the atomic model was refined using *REFMAC* (Winn *et al.*, 2003 ▸) and an omit difference map was examined using *Coot* (Emsley *et al.*, 2010 ▸) in the region where the ligands were expected to bind to the protein surface. The omit difference map was contoured at 1.5σ and displayed using *PyMOL* (DeLano, 2002 ▸). The omit maps calculated from the four-way clustering data were not observed to closely match any of the four lysozyme forms known to have been acoustically harvested onto the micro-meshes (data not shown). We concluded from this result that the clustering algorithm was not sufficiently sensitive to differentiate these four classes of very similar crystals using only variations in the observed unit-cell parameters. However, the omit maps calculated from the two-way clustering data were a good fit to the expected lysozyme forms (Fig. 2 ▸). We concluded from this result that the two-ligand form was sufficiently different from the native form that unit-cell-based clustering could be successful.

To perform the four-way split, we combined the universality advantage of cell-based clustering (Section 3) with the granularity advantage of intensity-based clustering (Section 4).

## 3. Clustering on unit-cell parameters

Stills and wedges of very low completeness are more appropriate for unit-cell parameter clustering, rather than reflection clustering, because pairs of images with very few commensurate reflections may still provide reasonable estimates of unit cells but may not provide enough data to compute a meaningful distance between sets of reflections.

The default *BLEND* approach to clustering on unit-cell parameters is to use (*a*, *b*, *c*, α, β, γ) as a six-value vector, perform a principal component analysis (PCA), drop the components without significant variance and use the Euclid­ean distance calculated from the remaining components. This approach does not deal as effectively with the discontinuities produced by experimental error and ambiguities in reduction (for example between type I and type II cells and near cubic unit cells) as does the Andrews–Bernstein Niggli-cone distance (NCDist) algorithm (Andrews & Bernstein, 2014 ▸). NCDist allows slightly larger clusters of truly similar data sets to be formed, working in *G* ^6^ space, which uses Niggli reduction in a six-dimensional space consisting of the metric tensor with the last three components doubled: [*a* ^2^, *b* ^2^, *c* ^2^, 2*bc* cos(α), 2*ac* cos(β), 2*ab* cos(γ)].

**Figure 3:**
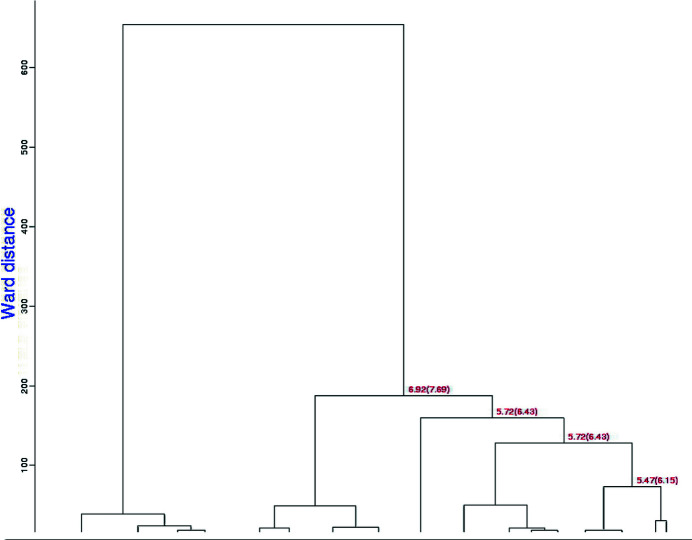
This dendrogram presents the top levels of *BLEND* clustering using the original less-sensitive *BLEND* unit-cell parameter distance function. The numbers are the LCV and the aLCV, with the aLCV in parentheses.

In our test case of 999 data sets of lysozyme with NAG and benzamidine soaks, 998 clusters were found with a completeness ranging from 40% to 100%. The top levels of the two dendrograms are shown in Figs. 3 ▸ and 4 ▸. The clusters are labeled by linear cell variation (LCV), which measures the maximum linear increase or decrease of the diagonals on the three independent cell faces (Foadi *et al.*, 2013 ▸). Values below 1% generally indicate a good degree of isomorphism among different crystals. Structural differences start to be noticeable with an LCV greater than 1.5%. A value in ångströms associated with the LCV is provided by the absolute linear cell variation (aLCV). Note the smaller Ward distances, *i.e.* tighter clusters, for the equivalent clusters in the latter NCDist-based dendrogram compared with the former.

The dendrograms are qualitatively similar, but for these test data the discrimination of the clustering changes. Using the original *BLEND* algorithm, the largest clusters that are 100% native, 100% NAG, 100% benzamidine and 100% benz­amidine plus NAG contain four, 15, five and ten data sets, respectively. Using NCDist clustering, the largest clusters that are 100% native, 100% NAG, 100% benzamidine and 100% benzamidine plus NAG contain nine, 15, eight and seven data sets, respectively. This provides a better base for switching over from cell clustering to reflection clustering; half of the 100% pure clusters are larger using NCDist.

## 4. Clustering on reflections

In a regime of high completeness, say 90%, different data sets can have enough reflections with common *hkl* indices to generate a satisfactory similarity or distance for clustering. If the data have been scaled, an *R* value can be used as a distance, but for unscaled data the preferred approach is to use a Pearson correlation coefficient (CC) as a measure of similarity, *i.e.* with a larger value of the coefficient for sets of reflections that are similar and a smaller value of the coefficient for sets of reflections that are dissimilar. The Pearson correlation coefficient is essentially the cosine of the angle between vectors of data. The lack of common scaling is dealt with by subtracting the mean (μ) of each vector from each component and dividing by the norm (||.||) of each to obtain two unit length vectors. Recall that the dot product (·) of two vectors is equal to the product of the norms of the two vectors times the cosine of the angle between them. 

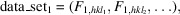




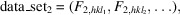




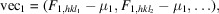




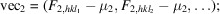




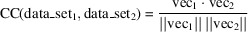

In order to extend the range of applicability of CC, we convert it to a distance, 

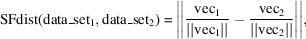

which is related to CC by 

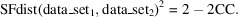

This choice of SFDist allows the distance to be adjusted to account for the greater uncertainty in cases where a pair of data sets has few common reflections, less than 90%, for example, by adding a penalty to the distance for each unmatched reflection.

## 5. Impact of choices in clustering

**Figure 5:**
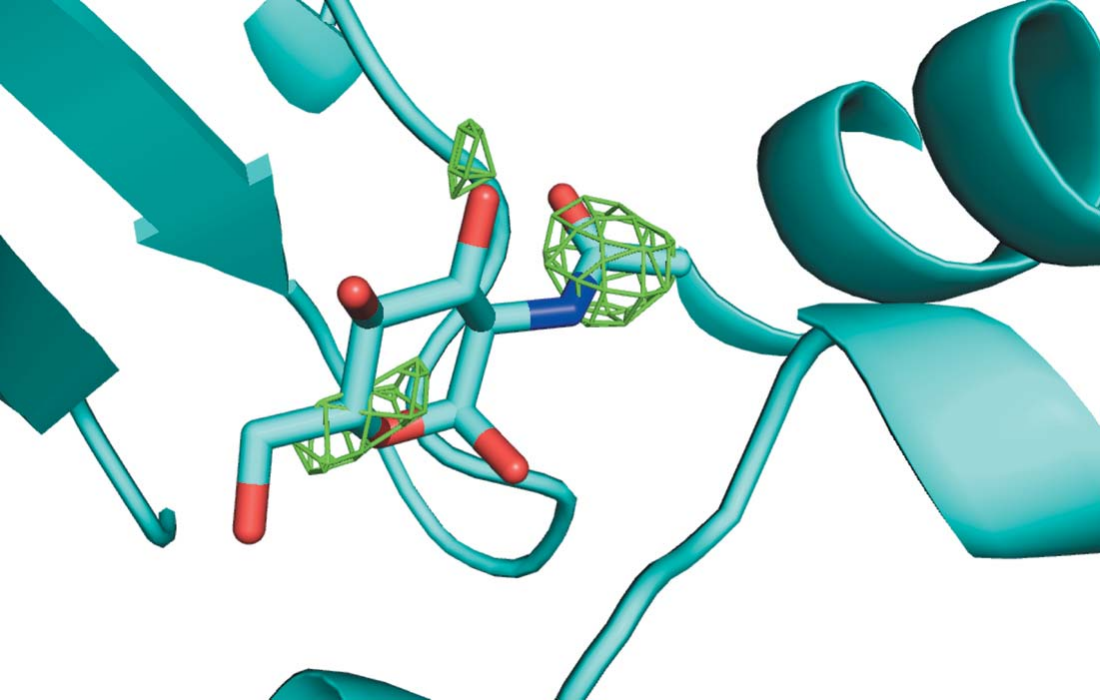
Omit difference map of the NAG site in cluster 28 of a two-stage clustering with *KAMO* using unit-cell parameters and NCDist to reach 10% completeness and then CC clustering with SFDist.

**Figure 8:**
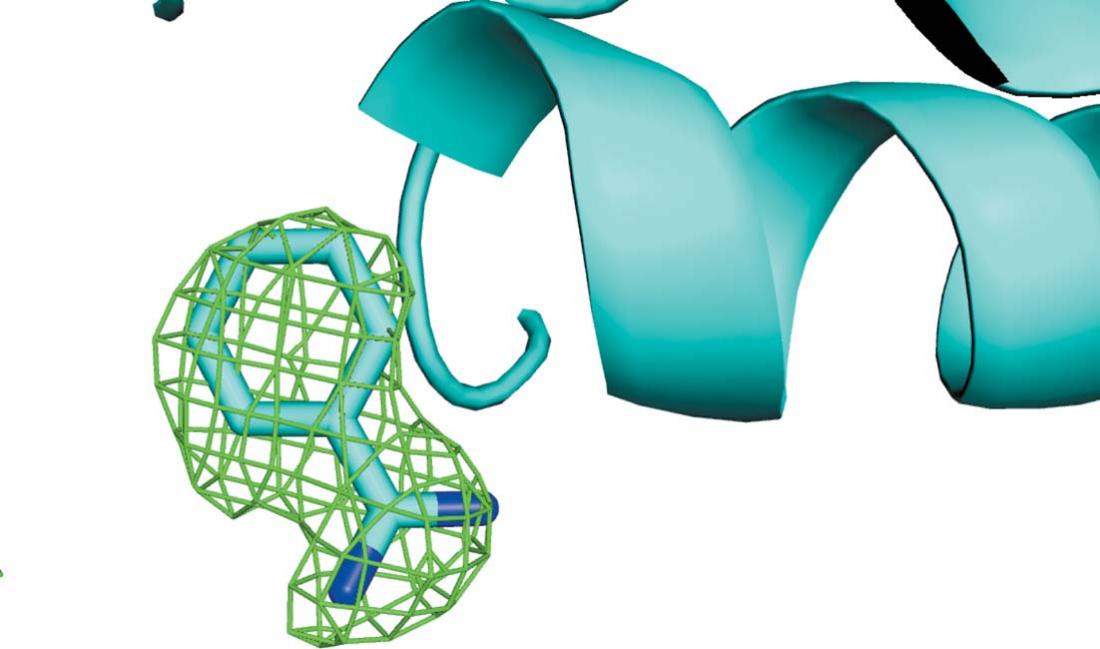
Omit difference map of the benzamidine site in cluster 28 of a two-stage clustering with *KAMO* using unit-cell parameters and NCDist to reach 10% completeness and then CC clustering with SFDist.

Unambiguous benzamidine-only, NAG-only and benzamidine plus NAG clusters are shown in the omit difference maps of the NAG site in clusters 28, 43 and 62 in Figs. 5 ▸, 6 ▸ and 7 ▸, respectively. Omit difference maps of the benzamidine site in clusters 28, 43 and 62 are shown in Figs. 8 ▸, 9 ▸ and 10 ▸, respectively. These are the results of two-stage *KAMO* clustering of the test data using NCDist unit-cell parameter-based clustering to reach 10% completeness and then SFDist reflection-based clustering of the resulting 107 non-overlapping clusters. For an example of this approach when using a higher level of completeness before the cutover from unit-cell parameter-based clustering to intensity-based clustering, see Nguyen *et al.* (2022 ▸).

**Figure 11:**
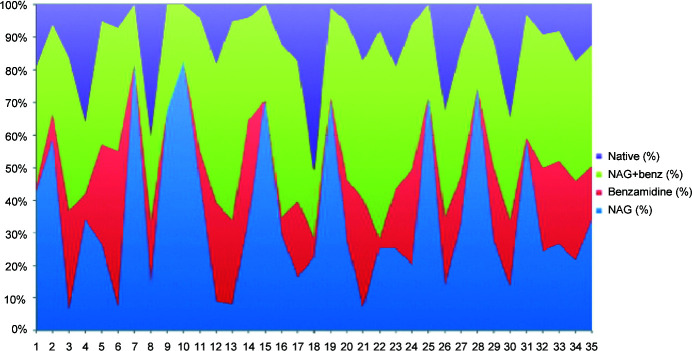
Color charts of the 35 largest data-set clusters for the NCDist clustering. From top to bottom the color blocks are the native soak, the benzamidine plus NAG soak, the benzamidine soak and the NAG soak. If one color reaches nearly from the bottom to the top at a given position then that cluster is a nearly pure species.

The impact of using clustering on reflections for larger clusters can be seen by looking at how well represented reasonably pure clusters are. In Figs. 11 ▸ and 12 ▸ we represent the purity of native, NAG, benzamidine and benzamidine plus NAG species using NCDist and SFDist.

The extreme variations in the SFDist results suggest two important lessons.

(i) It is best to use reflection-based clustering starting from data sets that are small enough to still be likely to be pure species, *i.e.* to use cell-based clustering only just far enough to reach a sufficient completeness that intensity-based clustering can be handled.
(ii) It is not necessarily desirable to continue clustering to the largest of the ‘best’ possible clusters. Smaller clusters of sufficient quality for processing are more likely to be pure species.

## 6. Discussion

Because microcrystals are expected to react quickly and uniformly to changes in their environment, serial crystallo­graphy is a desirable tool for examining the plasticity with which protein crystals respond to external perturbations. In some cases the external perturbation can be physical, such as conformational changes induced by light (Young *et al.*, 2016 ▸). In other cases proteins are perturbed by chemical means (Fromme, 2015 ▸). Often it is not possible to to draw a sharp boundary between diffraction images from different protein forms without the assistance of some type of clustering software. In response to this, many groups have developed effective clustering algorithms that use a measurable parameter from each diffraction still or wedge to cluster the data into categories which can then be merged to hopefully yield the electron density from a single protein isoform. Examples of measurable parameters that have been used for this purpose include unit-cell dimensions (Foadi *et al.*, 2013 ▸; Zeldin *et al.*, 2015 ▸) and diffraction intensities (Assmann *et al.*, 2016 ▸; Diederichs, 2017 ▸). What is striking about many of these physical parameters is that they are largely independent of one another.

Consequently, it should be possible to greatly improve the efficacy of data-clustering software by combining quasi-independent information in a multi-stage partitioning strategy (as presented here). An alternative that one might consider in some cases would be to combine the same data in a single higher-dimensional (more independent parameters) stage. However, all clustering methods work increasingly poorly as the number of independent parameters increases due to the ‘curse of dimensionality’ (Bellman, 1956 ▸). Combining very well behaved low-dimensionality cell-based clustering with high-dimensionality intensity-based clustering gives up the advantage of the reliability of cell-based clustering. In most cases, unless all the information to be gained from cell-based clustering is available *a priori*, it is probably best to take advantage of that information first and then move on to intensity-based clustering in a second stage, as we have done here.

We have demonstrated one possible approach to multi-stage data clustering. Our strategy was to use unit-cell-based clustering until the merged data were of sufficient completeness to then use intensity-based clustering. We have demonstrated that using this strategy we were able to accurately cluster data sets from crystals that had subtle differences. Certainly if one is dealing with a case in which the ‘correct’ symmetry and indexing of all reflections are known for all images, it makes sense to perform only intensity-based clustering, but in the general case performing cell-based clustering first makes sense.

## 7. Availability of data

The HKL structure-factor files and *BLEND* clustering data files used for the final intensity-based clustering are available from Zenodo (https://doi.org/10.5281/zenodo.6558532). The data sets were collected on the Highly Automated Macromolecular Crystallography (AMX) beamline 17-ID-1 at National Synchrotron Light Source II (NSLS-II) (Fuchs *et al.*, 2016 ▸). The coordinates have been deposited in the Protein Data Bank (Bernstein *et al.*, 1977 ▸; Berman *et al.*, 2000 ▸) as PDB entries 8dct (lysozyme from cluster 0003, double apo), 8dcu (lysozyme from cluster 0028, benzamidine ligand), 8dcv (lysozyme from cluster 0043, NAG ligand) and 8dcw (lysozyme from cluster 0062, NAG and benzamidine ligands).

## 8. Related literature

The following references are cited in the supporting information for this article: Kleywegt & Jones (1997 ▸) and Soares & Caspar (2017 ▸). 

## Supplementary Material

PDB reference: lysozyme, apo, 8dct

PDB reference: complex with benzamidine, 8dcu

PDB reference: complex with NAG, 8dcv

PDB reference: complex with NAG and benzamidine, 8dcw

The HKL structure-factor files and BLEND clustering data files used for the final intensity-based clustering: https://doi.org/10.5281/zenodo.6558532

Electron counting of ligand occupancies, data-reduction and structure-solving statistica, Supplementary Figures and Supplementary Tables. DOI: 10.1107/S2053230X22006422/ow5032sup1.pdf

## Figures and Tables

**Figure 4:**
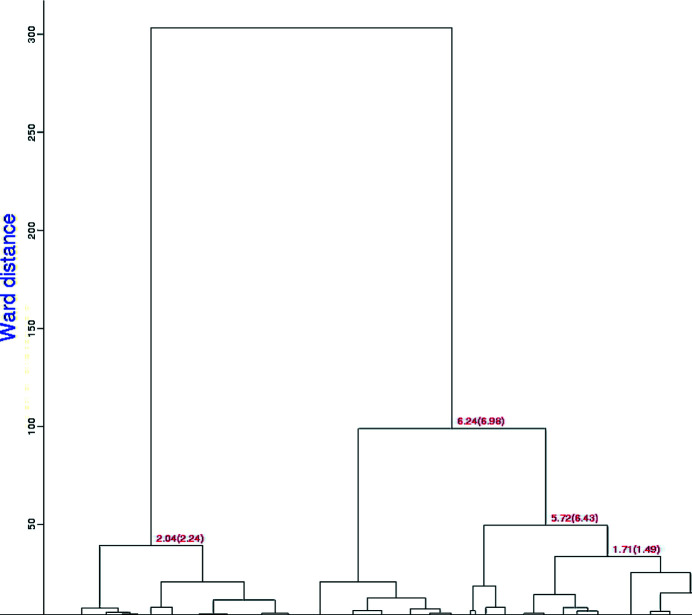
This dendrogram presents the top levels of *BLEND* clustering using the more sensitive Andrews–Bernstein Niggli-cone distance (NCDist) algorithm. The numbers are the LCV and the aLCV, with the aLCV in parentheses. Clustering is guided by the progressive merging of separate clusters into larger clusters using a measure of cluster proximity known as the Ward distance. This is equal to the increase of the distance variance (between each element of a cluster and its centroid) resulting from the merging of two separate clusters (Ward, 1963 ▸). Note that the Ward distances are smaller than those for the equivalent clusters in Fig. 3 ▸.

**Figure 6:**
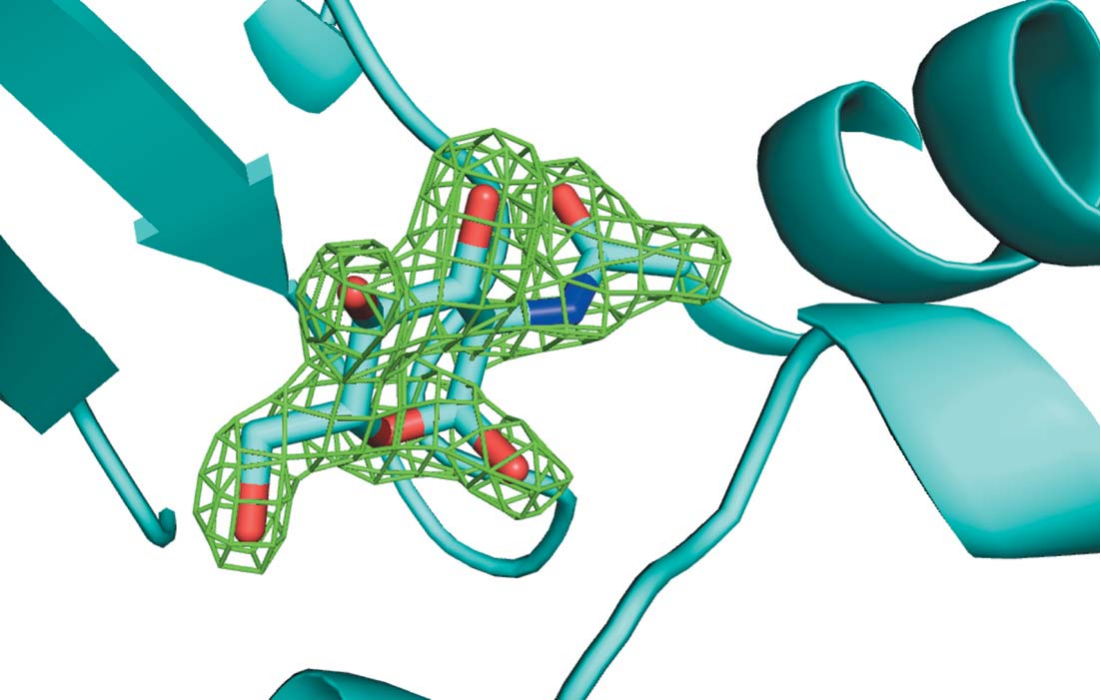
Omit difference map of the NAG site in cluster 43 of a two-stage clustering with *KAMO* using unit-cell parameters and NCDist to reach 10% completeness and then CC clustering with SFDist.

**Figure 7:**
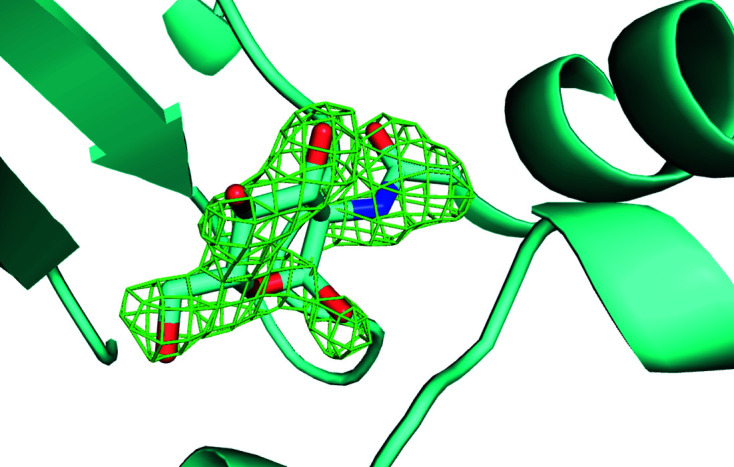
Omit difference map of the NAG site in cluster 62 of a two-stage clustering with *KAMO* using unit-cell parameters and NCDist to reach 10% completeness and then CC clustering with SFDist.

**Figure 9:**
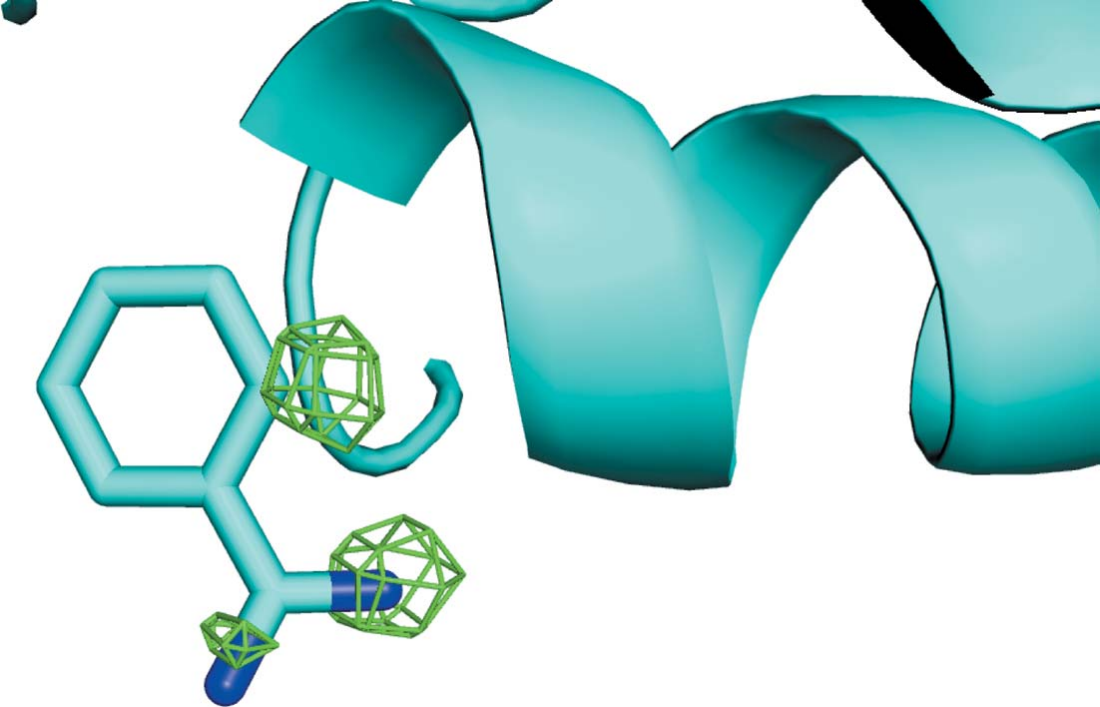
Omit difference map of the benzamidine site in cluster 43 of a two-stage clustering with *KAMO* using unit-cell parameters and NCDist to reach 10% completeness and then CC clustering with SFDist.

**Figure 10:**
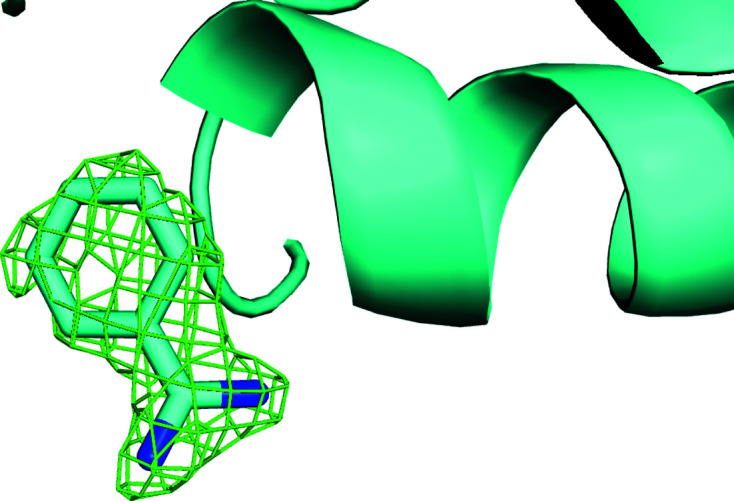
Omit difference map of the benzamidine site in cluster 62 of a two-stage clustering with *KAMO* using unit-cell parameters and NCDist to reach 10% completeness and then CC clustering with SFDist.

**Figure 12:**
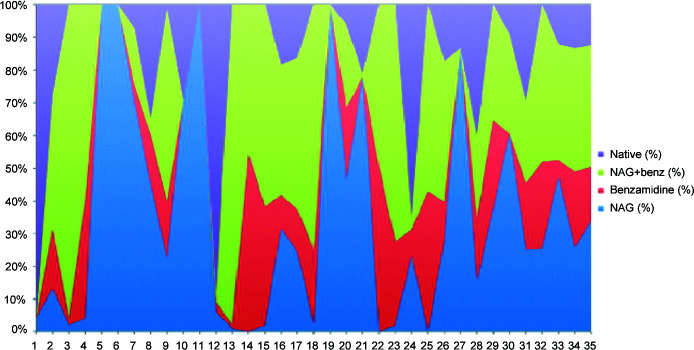
Color charts of the 35 largest data-set clusters for the SFDist clustering. From top to bottom the color blocks are the native soak, the benzamidine plus NAG soak, the benzamidine soak and the NAG soak. If one color reaches nearly from the bottom to the top at a given position then that cluster is a nearly pure species. This is the case for each soak at the left end of this SFDist chart.

## References

[bb1] Andrews, L. C. & Bernstein, H. J. (2014). *J. Appl. Cryst.* **47**, 346–359. 10.1107/S1600576713031002 PMC3937813 24587789

[bb2] Assmann, G., Brehm, W. & Diederichs, K. (2016). *J. Appl. Cryst.* **49**, 1021–1028. 10.1107/S1600576716005471 PMC4886987 27275144

[bb3] Bellman, R. (1956). *Dynamic Programming.* Santa Monica: The Rand Corporation.

[bb4] Berman, H. M., Westbrook, J., Feng, Z., Gilliland, G., Bhat, T. N., Weissig, H., Shindyalov, I. N. & Bourne, P. E. (2000). *Nucleic Acids Res.* **28**, 235–242. 10.1093/nar/28.1.235 PMC102472 10592235

[bb5] Bernstein, F. C., Koetzle, T. F., Williams, G. J. B., Meyer, E. F. Jr, Brice, M. D., Rodgers, J. R., Kennard, O., Shimanouchi, T. & Tasumi, M. (1977). *J. Mol. Biol.* **112**, 535–542. 10.1016/s0022-2836(77)80200-3 875032

[bb6] Brehm, W. & Diederichs, K. (2014). *Acta Cryst.* D**70**, 101–109. 10.1107/S1399004713025431 24419383

[bb7] Cole, K., Roessler, C. G., Mulé, E. A., Benson-Xu, E. J., Mullen, J. D., Le, B. A., Tieman, A. M., Birone, C., Brown, M., Hernandez, J., Neff, S., Williams, D., Allaire, M., Orville, A. M., Sweet, R. M. & Soares, A. S. (2014). *PLoS One*, **9**, e101036. 10.1371/journal.pone.0101036 PMC4079544 24988328

[bb8] Colella, R. & Luccio, A. (1984). *Opt. Commun.* **50**, 41–44.

[bb9] Cuttitta, C. M., Ericson, D. L., Scalia, A., Roessler, C. G., Teplitsky, E., Joshi, K., Campos, O., Agarwal, R., Allaire, M., Orville, A. M., Sweet, R. M. & Soares, A. S. (2015). *Acta Cryst.* D**71**, 94–103. 10.1107/S1399004714013728 PMC4304690 25615864

[bb10] DeLano, W. L. (2002). *CCP4 Newsl. Protein Crystallogr.* **40**, 82–92.

[bb11] Diederichs, K. (2017). *Acta Cryst.* D**73**, 286–293. 10.1107/S2059798317000699 PMC5379934 28375141

[bb12] Emsley, P., Lohkamp, B., Scott, W. G. & Cowtan, K. (2010). *Acta Cryst.* D**66**, 486–501. 10.1107/S0907444910007493 PMC2852313 20383002

[bb14] Foadi, J., Aller, P., Alguel, Y., Cameron, A., Axford, D., Owen, R. L., Armour, W., Waterman, D. G., Iwata, S. & Evans, G. (2013). *Acta Cryst.* D**69**, 1617–1632. 10.1107/S0907444913012274 PMC3727331 23897484

[bb15] Foley, B. J., Drozd, A. M., Bollard, M. T., Laspina, D., Podobedov, N., Zeniou, N., Rao, A. S., Andi, B., Jackimowicz, R., Sweet, R. M., McSweeney, S. & Soares, A. S. (2016). *SLAS Technol.* **21**, 115–124. 10.1177/2211068215616072 26564917

[bb16] Fromme, P. (2015). *Nat. Chem. Biol.* **11**, 895–899. 10.1038/nchembio.1968 PMC4839532 26575227

[bb17] Fuchs, M. R., Bhogadi, D. K., Jakoncic, J., Myers, S., Sweet, R. M., Berman, L. E., Skinner, J., Idir, M., Chubar, O., McSweeney, S. & Schneider, D. K. (2016). *AIP Conf. Proc.* **1741**, 030006.

[bb18] Gildea, R. J. & Winter, G. (2018). *Acta Cryst.* D**74**, 405–410. 10.1107/S2059798318002978 PMC5930348 29717711

[bb19] Giordano, R., Leal, R. M. F., Bourenkov, G. P., McSweeney, S. & Popov, A. N. (2012). *Acta Cryst.* D**68**, 649–658. 10.1107/S0907444912006841 22683787

[bb20] Hasegawa, K., Yamashita, K., Murai, T., Nuemket, N., Hirata, K., Ueno, G., Ago, H., Nakatsu, T., Kumasaka, T. & Yamamoto, M. (2017). *J. Synchrotron Rad.* **24**, 29–41. 10.1107/S1600577516016362 PMC5182019 28009544

[bb21] Kirian, R. A., Wang, X., Weierstall, U., Schmidt, K. E., Spence, J. C. H., Hunter, M., Fromme, P., White, T., Chapman, H. N. & Holton, J. (2010). *Opt. Express*, **18**, 5713–5723. 10.1364/OE.18.005713 PMC4038330 20389587

[bb22] Kleywegt, G. J. & Jones, T. A. (1997). *Methods Enzymol.* **277**, 208–230. 10.1016/s0076-6879(97)77013-7 18488311

[bb23] Liu, Q., Liu, Q. & Hendrickson, W. A. (2013). *Acta Cryst.* D**69**, 1314–1332. 10.1107/S0907444913001479 PMC3689535 23793158

[bb24] Neutze, R., Wouts, R., van der Spoel, D., Weckert, E. & Hajdu, J. (2000). *Nature*, **406**, 752–757. 10.1038/35021099 10963603

[bb25] Nguyen, T., Phan, K. L., Kozakov, D., Gabelli, S. B., Kreitler, D. F., Andrews, L. C., Jakoncic, J., Sweet, R. M., Soares, A. S. & Bernstein, H. J. (2022). *Acta Cryst.* D**78**, 268–277. 10.1107/S2059798321013425 PMC8900820 35234141

[bb26] Pearson, A. R. & Mehrabi, P. (2020). *Curr. Opin. Struct. Biol.* **65**, 168–174. 10.1016/j.sbi.2020.06.019 32846363

[bb27] Roessler, C. G., Agarwal, R., Allaire, M., Alonso-Mori, R., Andi, B., Bachega, J. F. R., Bommer, M., Brewster, A. S., Browne, M. C., Chatterjee, R., Cho, E., Cohen, A. E., Cowan, M., Datwani, S., Davidson, V. L., Defever, J., Eaton, B., Ellson, R., Feng, Y., Ghislain, L. P., Glownia, J. M., Han, G., Hattne, J., Hellmich, J., Héroux, A., Ibrahim, M., Kern, J., Kuczewski, A., Lemke, H. T., Liu, P., Majlof, L., McClintock, W. M., Myers, S., Nelsen, S., Olechno, J., Orville, A. M., Sauter, N. K. S., Soares, A., Soltis, S. M., Song, H., Stearns, R. G., Tran, R., Tsai, Y., Uervirojnangkoorn, M., Wilmot, C. M., Yachandra, V., Yano, J., Yukl, E. T., Zhu, D. & Zouni, A. (2016). *Structure*, **24**, 631–640.

[bb28] Rossmann, M. G. (2014). *IUCrJ*, **1**, 84–86. 10.1107/S2052252514000499 PMC4062086 25075323

[bb30] Soares, A. S. & Caspar, D. L. D. (2017). *J. Struct. Biol.* **200**, 213–218. 10.1016/j.jsb.2017.08.004 28838818

[bb31] Soares, A. S., Engel, M. A., Stearns, R., Datwani, S., Olechno, J., Ellson, R., Skinner, J. M., Allaire, M. & Orville, A. M. (2011). *Biochemistry*, **50**, 4399–4401. 10.1021/bi200549x PMC3144476 21542590

[bb32] Standfuss, J. & Spence, J. (2017). *IUCrJ*, **4**, 100–101. 10.1107/S2052252517001877 PMC5330517 28250945

[bb33] Thompson, M. C., Cascio, D. & Yeates, T. O. (2018). *Acta Cryst.* D**74**, 411–421. 10.1107/S2059798318003479 PMC5930349 29717712

[bb34] Ward, J. H. Jr (1963). *J. Am. Stat. Assoc.* **58**, 236–244.

[bb35] White, T. A., Kirian, R. A., Martin, A. V., Aquila, A., Nass, K., Barty, A. & Chapman, H. N. (2012). *J. Appl. Cryst.* **45**, 335–341.

[bb37] Winn, M. D., Murshudov, G. N. & Papiz, M. Z. (2003). *Methods Enzymol.* **374**, 300–321. 10.1016/S0076-6879(03)74014-2 14696379

[bb38] Yamashita, K. (2017). *Nihon Kessho Gakkaishi*, **59**, 207–208.

[bb39] Yamashita, K., Hirata, K., Kawano, Y., Ueno, G., Hasegawa, K., Kumasaka, T. & Yamamoto, M. (2017). *Acta Cryst.* A**73**, a335. 10.1107/S2059798318017795 PMC6400253 30821703

[bb40] Yamashita, K., Hirata, K. & Yamamoto, M. (2018). *Acta Cryst.* D**74**, 441–449. 10.1107/S2059798318004576 PMC5930351 29717715

[bb41] Yin, X., Scalia, A., Leroy, L., Cuttitta, C. M., Polizzo, G. M., Ericson, D. L., Roessler, C. G., Campos, O., Ma, M. Y., Agarwal, R., Jackimowicz, R., Allaire, M., Orville, A. M., Sweet, R. M. & Soares, A. S. (2014). *Acta Cryst.* D**70**, 1177–1189. 10.1107/S1399004713034603 PMC4014116 24816088

[bb42] Young, I. D., Ibrahim, M., Chatterjee, R., Gul, S., Fuller, F. D., Koroidov, S., Brewster, A. S., Tran, R., Alonso-Mori, R., Kroll, T., Michels-Clark, T., Laksmono, H., Sierra, R. G., Stan, C. A., Hussein, R., Zhang, M., Douthit, L., Kubin, M., de Lichtenberg, C., Vo Pham, L., Nilsson, H., Cheah, M. H., Shevela, D., Saracini, C., Bean, M. A., Seuffert, I., Sokaras, D., Weng, T.-C., Pastor, E., Weninger, C., Fransson, T., Lassalle, L., Bräuer, P., Aller, P., Docker, P. T., Andi, B., Orville, A. M., Glownia, J. M., Nelson, S., Sikorski, M., Zhu, D., Hunter, M. S., Lane, T. J., Aquila, A., Koglin, J. E., Robinson, J., Liang, M., Boutet, S., Lyubimov, A. Y., Uervirojnangkoorn, M., Moriarty, N. W., Liebschner, D., Afonine, P. V., Waterman, D. G., Evans, G., Wernet, P., Dobbek, H., Weis, W. I., Brunger, A. T., Zwart, P. H., Adams, P. D., Zouni, A., Messinger, J., Bergmann, U., Sauter, N. K., Kern, J., Yachandra, V. K. & Yano, J. (2016). *Nature*, **540**, 453–457.

[bb43] Zeldin, O. B., Brewster, A. S., Hattne, J., Uervirojnangkoorn, M., Lyubimov, A. Y., Zhou, Q., Zhao, M., Weis, W. I., Sauter, N. K. & Brunger, A. T. (2015). *Acta Cryst.* D**71**, 352–356. 10.1107/S1399004714025875 PMC4321488 25664746

