# Supplementary material for: Serial crystallography with multi-stage merging of thousands of images
Source: Acta Crystallogr F Struct Biol Commun. 2022 Jul 4;78(Pt 7):281–8. doi: 10.1107/S2053230X22006422 (PMC9254899; doi:10.1107/S2053230X22006422)
Supplement: Supplementary file 1 [file f-78-00281-sup1.pdf]

STRUCTURAL BIOLOGY
COMMUNICATIONS

Volume 78 (2022)

Supporting information for article:

Serial crystallography with multi-stage merging of thousands of images

Alexei S. Soares, Yusuke Yamada, Jean Jakoncic, Sean McSweeney, Robert M. Sweet, John Skinner, James Foadi, Martin R. Fuchs, Dieter K. Schneider, Wuxian Shi, Babak Andi, Lawrence C. Andrews and Herbert J. Bernstein

Supporting Information

S1. Electron Counting of Ligand Occupancies

The occupancy of benzamidine (BEN) and N-acetylglucosamine (NAG) ligands deduced from the integrated electron density in the volume occupied by each ligand is shown in Fig. S1. The data clusters 3 (Fig. S4), 28 (Fig. S5), 43 (Fig. S6), and 62 (Fig. S7) were obtained using two-stage clustering (BLEND^{NCDist} followed by BLEND^{SFDist}), as described in §4. Electron density maps were placed on an absolute scale using iterative density modification (Soares & Caspar, 2017) and the electron density within the envelope of each ligand was integrated. The occupancy was deduced by comparing the observed number of electrons within each ligand envelope with the expected number of electrons in the apo- and holo- structures. If we disregard any cross-contamination during sample preparation (§3, ¶3), we would expect perfect clustering to result in four electron density maps where all ligands have occupancy of zero or unity. The observed occupancies were 0.18 & 0.00 (native), 0.62 & 0.03 (BEN+/NAG-), 0.09 & 1.00 (BEN-/NAG+) and 1.00 & 0.67 (BEN+/NAG+). The observed occupancies are, on average, 12.6% different from the ideal values of zero and unity.

S2. Data Reduction and Structure Solving Statistics

The data reduction and structure solving statistics are given in Tables S1, S2 and S3. Because the largest clusters merge structures with significantly different ligands some of the R-factors degrade rather than improve. This is only to be expected. See (Kleywegt & Jones, 1997) for more information on R_{free} and R_{work}.

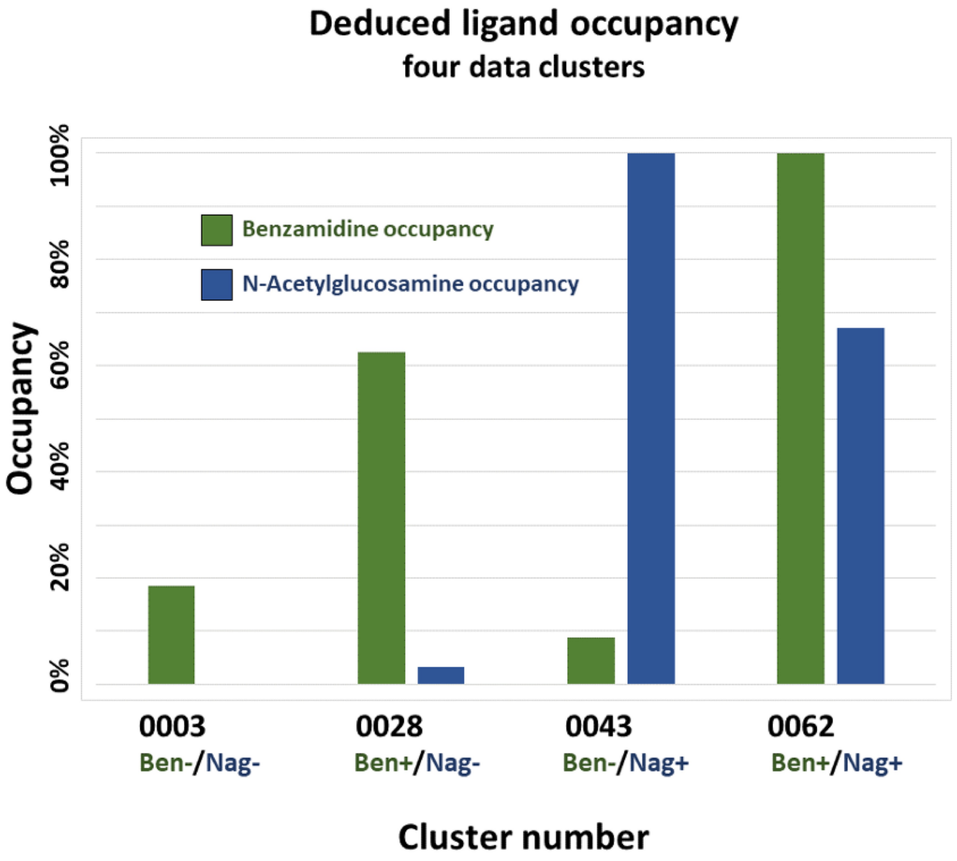

Fig. S1. Occupancy of benzamidine (BEN) and N-Acetylglucosamine (NAG) ligands deduced from the integrated electron density in the volume occupied by each ligand. The data clusters were obtained using two-stage clustering (BLEND^{NCDist} followed by BLEND^{SFDist})

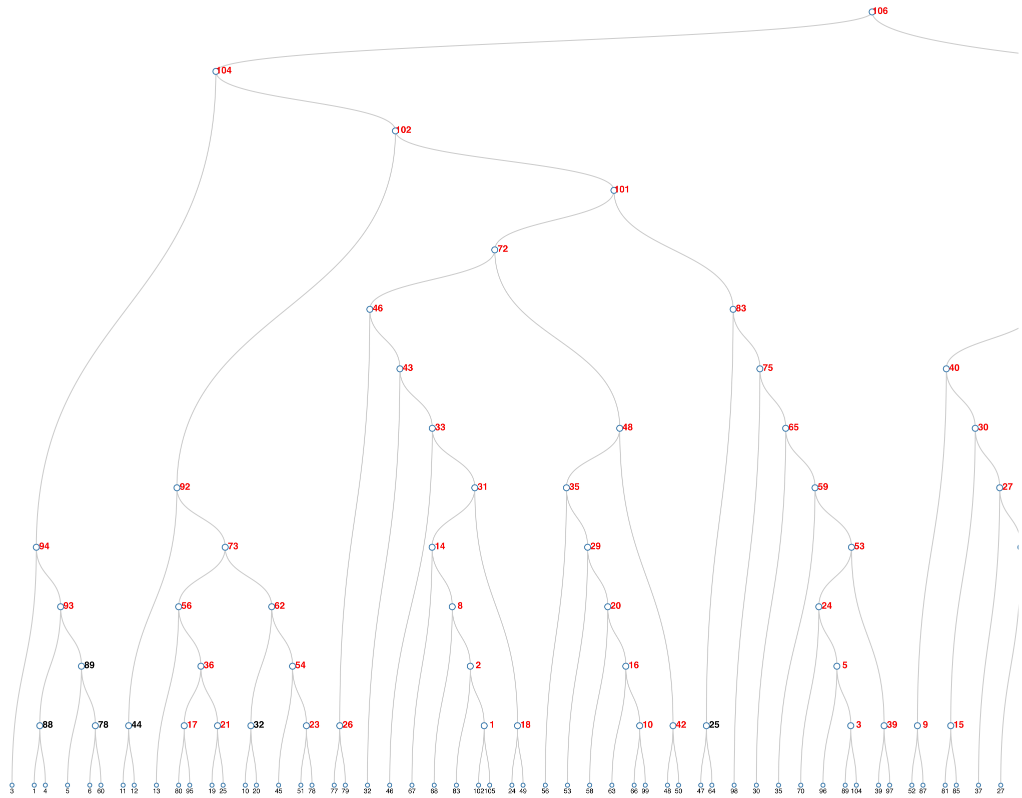

Fig. S2. Left half of the complete dendrogram for all the data clusters that were obtained using two-stage clustering (BLEND^{NCDist} followed by BLEND^{SFDist}). The other half of this dendrogram is shown in Fig. S3.

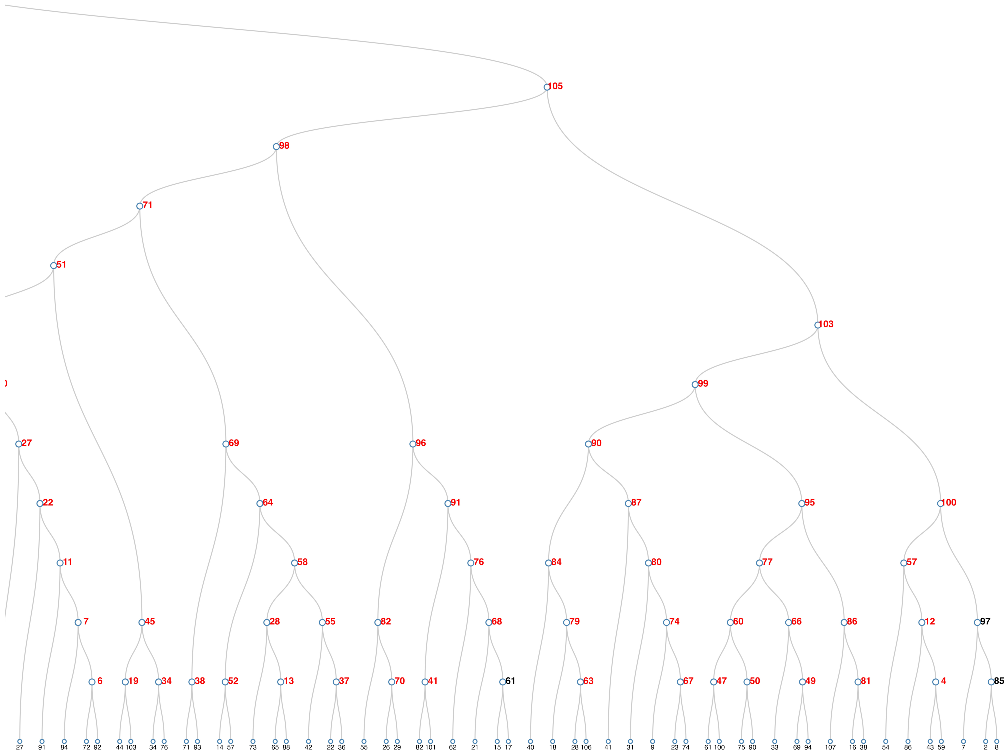

Fig. S3. Right half of the complete dendrogram for all the data clusters that were obtained using two-stage clustering (BLEND^{NCDist} followed by BLEND^{SFDist}). The other half of this dendrogram is shown in Fig. S2.

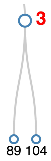

Fig. S4. Portion of the dendrogram in Figs. S2 and S3 showing cluster 3

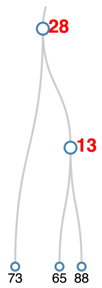

Fig. S5. Portion of the dendrogram in Figs. S2 and S3 showing cluster 28

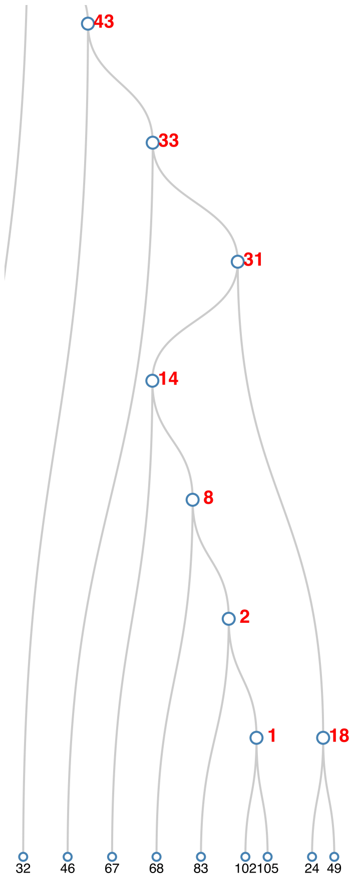

Fig. S6. Portion of the dendrogram in Figs. S2 and S3 showing cluster 43

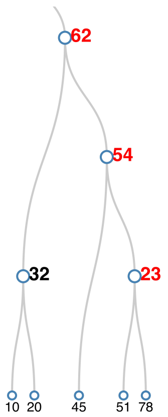

Fig. S7. Portion of the dendrogram in Figs. S2 and S3 showing cluster 62

Table S1. *Data reduction and structure solving statistics. Data are shown for all clusters determined using two factor clustering (NCDist followed by SFDist), continued in Tables S2 and S3*

Cluster Number	Rmerge (%)	Comp. (%)	RefNum	R_{work} Overall	R_{free} Overall	R_{work} Last shell	R_{free} Last shell	CC_{work} Overall	CC_{free} Overall
001	19.80	97.12	7740	0.172	0.230	0.204	0.266	0.950	0.916
002	22.60	99.19	7844	0.169	0.218	0.197	0.263	0.952	0.920
003	36.00	98.28	7909	0.212	0.286	0.345	0.349	0.946	0.904
004	19.30	92.99	7337	0.201	0.258	0.223	0.307	0.952	0.927
005	39.50	99.44	8004	0.208	0.282	0.331	0.336	0.945	0.899
006	28.50	96.13	7826	0.204	0.271	0.357	0.265	0.952	0.915
007	31.70	98.99	8059	0.196	0.253	0.327	0.345	0.953	0.930
008	23.00	99.62	7942	0.162	0.216	0.187	0.262	0.955	0.927
009	35.90	93.97	7580	0.235	0.315	0.368	0.333	0.935	0.887
010	28.70	98.60	7875	0.203	0.266	0.223	0.321	0.925	0.888
011	38.00	99.57	8108	0.192	0.249	0.324	0.321	0.953	0.930
012	33.80	98.10	7744	0.201	0.258	0.286	0.309	0.950	0.925
013	39.10	95.42	7794	0.223	0.286	0.349	0.405	0.929	0.891
014	25.40	99.83	7926	0.161	0.214	0.180	0.215	0.954	0.925
015	38.80	95.85	7851	0.220	0.287	0.307	0.308	0.937	0.902
016	34.70	99.36	7920	0.200	0.263	0.217	0.281	0.927	0.888
017	25.60	96.77	7770	0.179	0.235	0.236	0.299	0.944	0.921
018	30.20	90.16	7158	0.185	0.249	0.249	0.300	0.941	0.898
019	44.30	96.72	7800	0.234	0.296	0.332	0.384	0.936	0.896
020	32.40	99.85	7963	0.187	0.258	0.179	0.241	0.937	0.895
021	16.70	91.34	7298	0.173	0.247	0.275	0.309	0.952	0.914
022	38.20	99.74	8123	0.188	0.247	0.327	0.327	0.955	0.934
023	28.20	91.79	7371	0.187	0.247	0.220	0.311	0.936	0.909
024	41.80	99.72	8039	0.209	0.281	0.332	0.348	0.942	0.894
026	21.50	91.55	7286	0.173	0.240	0.212	0.246	0.946	0.899
027	39.80	99.83	8130	0.186	0.243	0.312	0.297	0.955	0.936
028	42.60	97.78	7988	0.212	0.271	0.352	0.403	0.940	0.915
029	36.10	99.85	7983	0.189	0.257	0.180	0.259	0.934	0.889
030	43.10	99.94	8140	0.178	0.233	0.264	0.282	0.956	0.938
031	27.90	99.89	7933	0.159	0.212	0.177	0.216	0.954	0.926
033	27.10	99.95	7938	0.157	0.209	0.164	0.214	0.954	0.928
034	54.40	94.52	7685	0.235	0.297	0.376	0.396	0.930	0.885
035	38.50	99.87	7985	0.196	0.266	0.190	0.251	0.929	0.880
036	24.40	99.25	7949	0.167	0.212	0.211	0.257	0.950	0.935
037	45.50	83.97	6854	0.223	0.311	0.331	0.505	0.946	0.904
038	48.50	96.99	7990	0.260	0.316	0.402	0.386	0.919	0.890
039	44.20	96.51	7770	0.231	0.306	0.353	0.291	0.930	0.879
040	43.40	99.97	8141	0.181	0.228	0.267	0.317	0.954	0.942
041	60.10	96.72	7938	0.259	0.345	0.362	0.421	0.901	0.829
042	27.60	91.75	7315	0.200	0.271	0.268	0.325	0.932	0.884
043	28.10	99.98	7940	0.159	0.210	0.161	0.212	0.953	0.925
045	56.40	99.58	8097	0.214	0.270	0.353	0.358	0.944	0.916
046	28.40	99.98	7940	0.158	0.210	0.167	0.225	0.954	0.927
047	51.10	97.24	7842	0.278	0.362	0.390	0.370	0.917	0.851

Table S2. *Data reduction and structure solving statistics. Data are shown for all clusters determined using two factor clustering (NCDist followed by SFDist), continued from Table S1 and continued in Table S3*

Cluster Number	Rmerge (%)	Comp. (%)	RefNum	R_{work} Overall	R_{free} Overall	R_{work} Last shell	R_{free} Last shell	CC_{work} Overall	CC_{free} Overall
048	40.70	99.98	7972	0.193	0.267	0.183	0.258	0.930	0.883
049	46.50	96.21	7771	0.272	0.350	0.416	0.404	0.910	0.842
050	52.70	96.94	7834	0.273	0.350	0.359	0.432	0.908	0.848
051	48.60	99.97	8141	0.177	0.230	0.253	0.309	0.955	0.939
052	40.30	92.89	7586	0.233	0.310	0.381	0.407	0.932	0.876
053	49.60	99.92	8053	0.204	0.275	0.297	0.296	0.944	0.898
054	39.80	97.44	7861	0.212	0.278	0.269	0.395	0.918	0.882
055	49.40	95.61	7812	0.222	0.304	0.343	0.446	0.945	0.909
056	24.90	99.50	7940	0.163	0.214	0.197	0.267	0.951	0.933
057	38.40	98.92	7810	0.204	0.261	0.273	0.337	0.948	0.923
058	54.80	99.63	8137	0.206	0.265	0.341	0.400	0.942	0.919
059	52.10	99.98	8052	0.202	0.276	0.282	0.296	0.942	0.895
060	55.80	99.57	8049	0.261	0.349	0.387	0.426	0.925	0.860
062	40.80	99.28	7919	0.193	0.264	0.230	0.340	0.935	0.901
063	48.70	96.83	7875	0.314	0.408	0.348	0.479	0.877	0.789
064	57.90	99.91	8165	0.202	0.259	0.333	0.370	0.941	0.920
065	53.20	99.99	8039	0.204	0.276	0.288	0.334	0.942	0.898
066	50.70	98.52	7985	0.270	0.350	0.404	0.366	0.914	0.849
067	49.90	96.12	7726	0.299	0.364	0.290	0.388	0.888	0.853
068	59.40	92.95	7526	0.257	0.338	0.388	0.384	0.914	0.857
069	61.50	99.92	8166	0.201	0.259	0.324	0.371	0.938	0.918
070	61.50	87.62	7190	0.291	0.363	0.403	0.233	0.897	0.833
071	54.10	99.92	8166	0.175	0.228	0.233	0.247	0.954	0.937
072	43.10	99.99	7941	0.181	0.234	0.185	0.285	0.941	0.905
073	36.10	99.99	7976	0.168	0.234	0.194	0.283	0.946	0.918
074	62.40	97.86	7855	0.289	0.363	0.315	0.404	0.893	0.853
075	55.90	99.99	8039	0.204	0.276	0.284	0.336	0.940	0.893
076	63.00	97.94	7931	0.261	0.340	0.376	0.328	0.914	0.854
077	67.00	99.98	8107	0.280	0.335	0.381	0.338	0.904	0.872
079	89.50	99.00	7974	0.377	0.417	0.440	0.501	0.740	0.710
080	64.40	98.94	7941	0.291	0.362	0.325	0.384	0.896	0.847
082	59.70	98.13	8053	0.281	0.369	0.363	0.424	0.899	0.845
083	56.60	99.99	8039	0.206	0.276	0.267	0.340	0.936	0.891
084	78.80	99.49	8014	0.372	0.411	0.395	0.424	0.790	0.760
086	64.20	98.45	7904	0.313	0.372	0.415	0.452	0.868	0.810
087	69.30	99.52	7988	0.290	0.364	0.328	0.392	0.896	0.847
090	90.30	99.89	8020	0.358	0.408	0.367	0.472	0.791	0.737
091	71.00	99.75	8079	0.249	0.341	0.353	0.378	0.911	0.846
092	35.20	99.99	7976	0.165	0.229	0.188	0.241	0.948	0.919
093	36.20	90.95	7230	0.225	0.296	0.334	0.341	0.923	0.863
094	36.50	92.16	7324	0.221	0.292	0.319	0.338	0.923	0.864
095	64.00	99.95	8105	0.267	0.354	0.392	0.355	0.916	0.855
096	74.70	99.91	8092	0.243	0.329	0.349	0.386	0.915	0.853

Table S3. *Data reduction and structure solving statistics. Data are shown for all clusters determined using two factor clustering (NCDist followed by SFDist), continued from Tables S1 and S2*

Cluster Number	Rmerge (%)	Comp. (%)	RefNum	R_{work} Overall	R_{free} Overall	R_{work} Last shell	R_{free} Last shell	CC_{work} Overall	CC_{free} Overall
098	63.80	99.92	8166	0.183	0.232	0.217	0.234	0.947	0.932
099	92.50	99.89	8020	0.336	0.386	0.384	0.458	0.802	0.764
100	48.60	99.28	7740	0.210	0.270	0.283	0.357	0.943	0.911
101	58.20	99.99	7941	0.203	0.254	0.184	0.258	0.919	0.883
102	59.70	99.99	7976	0.204	0.269	0.190	0.286	0.921	0.870
103	96.60	99.93	7790	0.303	0.380	0.394	0.416	0.820	0.736
104	61.70	99.99	7946	0.208	0.270	0.187	0.285	0.916	0.868
105	92.60	99.93	7790	0.247	0.317	0.272	0.359	0.859	0.793
106	88.60	99.99	7946	0.242	0.315	0.261	0.343	0.855	0.764
